# Molecular identification of *Clonorchis sinensis *and discrimination with other opisthorchid liver fluke species using multiple Ligation-depended Probe Amplification (MLPA)

**DOI:** 10.1186/1756-3305-4-98

**Published:** 2011-06-07

**Authors:** Jiufeng Sun, Jin Xu, Pei Liang, Qiang Mao, Yan Huang, Xiaoli Lv, Chuanhuan Deng, Chi Liang, G S de Hoog, Xinbing Yu

**Affiliations:** 1 Department of Parasitology, Zhongshan School of Medicine; Key Laboratory for Tropical Diseases Control, Ministry of Education, Sun Yat-sen University. No 74, The Second Zhongshan RD, Guangzhou, Guangdong, 510080, China; 2 Institute for Biodiversity and Ecosystem Dynamics, University of Amsterdam, The Netherlands

## Abstract

**Background:**

Infections with the opisthorchid liver flukes *Clonorchis sinensis*, *Opisthorchis viverrini*, and *O. felineus *cause severe health problems globally, particularly in Southeast Asia. Early identification of the infection is essential to provide timely and appropriate chemotherapy to patients.

**Results:**

In this study we evaluate a PCR-based molecular identification method, Multiplex Ligation-dependent Probe Amplification (MLPA), which allows rapid and specific detection of single nucleotide acid differences between *Clonorchis sinensis*, *Opisthorchis viverrini *and *O. felineus*. Three probe pairs were derived from the Internally Transcribed Spacer 1 (ITS1) of three opisthorchid liver flukes using a systematic phylogenetic analysis. Specific loci were detected in all three species, yielding three amplicons with 198,172 and 152 bp, respectively, while no cross reactions were observed. A panel of 66 *C. sinensis *isolates was screened using MLPA. All species were positively identified, and no inhibition was observed. The detection limit was 10^3 ^copies of the ITS gene for the three liver flukes, or about 60 pg genomic DNA for *Clonorchis sinensis*. Amplification products can be detected by electrophoresis on agarose gel or in a capillary sequencer. In addition, genomic DNA of *Clonorchis sinensis *in fecal samples of infected rats was positively amplified by MLPA.

**Conclusion:**

The flexibility and specificity make MLPA a potential tool for specific identification of infections by opisthorchid liver flukes in endemic areas.

## Background

*Clonorchis sinensis *(*C. sinensis*), *Opisthorchis viverrini *(*O. viverrini*) and *Opisthorchis felineus *(*O. felineus*) (Opisthorchiidae) are among the most frequent endemic food-borne liver flukes, causing severe clonorchiasis and opisthorchiasis. Humans contract the disease through consumption of raw or inadequately cooked freshwater fish containing the infective metacercariae. About 35 million people are infected with *C. sinensis *globally. Main endemic areas are located in southern Asia including China, Korea, Japan, Taiwan and Vietnam [1]. In China the estimated infection by *C. sinensis *is 15 million [2,3]. Approximately 9 million people are infected with *O. viverrini *in Thailand, Cambodia, and Laos [4-6]. In eastern Europe 1.2 to 1.5 million patients are infected with *O. felineus *[7]. In recent years, endemic areas of liver flukes are expanding to North America and Europe due to fish import and immigration [7-9].

Current clinical diagnosis of liver fluke infection is by direct microscopy of eggs in feces. However, this procedure is time-consuming and inaccurate, resulting in false-negatives due to the difficulty to distinguish eggs from each other or from those of closely related heterophyides [10-12]. As a result, appropriate chemotherapy may be delayed. Hence there is an urgent need for a novel tool to diagnose the infection.

A number of methods have been developed to identify or detect liver flukes using DNA, mRNA or protein. Among PCR-based molecular methods, nested-PCR [13] and loop-mediated isothermal amplification (LAMP) [14] are particularly promising [15-19]. However, simple PCR amplification carries the risk of false-negative data, due to PCR inhibitors involved in complex samples [20,21]. Moreover, mixed liver fluke infection may also hamper the application of simple PCR [22]. As an alternative, multiplex PCR may achieve high efficiency with simultaneous amplification of two or more genetic loci in one reaction, while this also may reduce the number of false-negative or false-positive results [23]. Although multiplex PCR has been reported to discriminate between C. *sinensis *and *O. viverrini *[24], it is technically difficult to optimize PCRs for amplification of multiple genes or loci, because each primer pair requires a different optimal combination of reagents and annealing temperatures. To overcome this problem we developed a multiplex PCR amplification technique using multiplex ligation-dependent probe amplification (MLPA).

**Figure 1:**
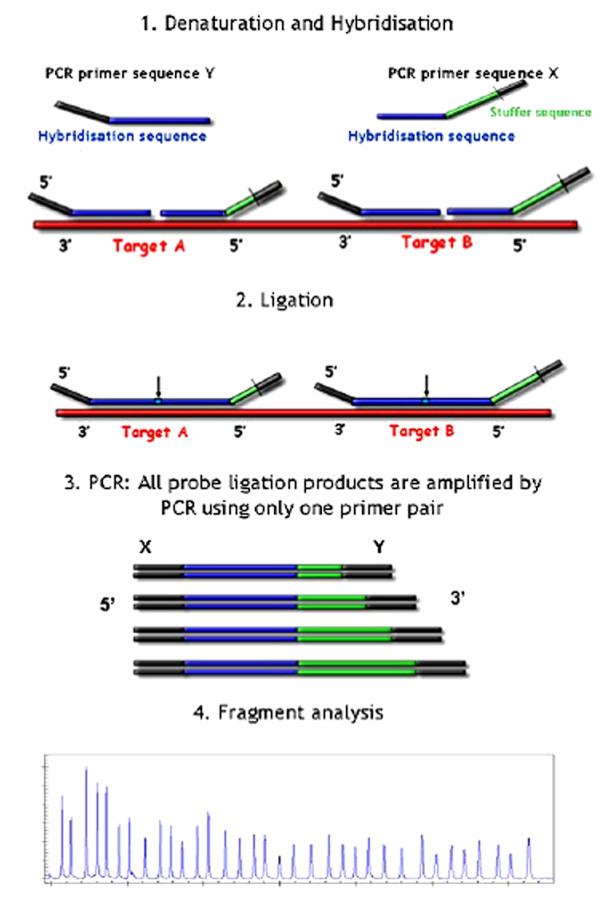
**Outline of the MLPA technique (http://www.mlpa.com)**. After hybridisation to their target sequence in the sample DNA, the probe oligonucleotides are enzymatically ligated. One probe oligonucleotide contains a non-hybridising stuffer sequence of variable length. Ligation products can be amplified using PCR primer sequences X and Y amplification product of each probe has a unique length (130-480 nt). Amplification products are separated by electrophoresis.

MLPA is a simple, robust and fast method designed for simultaneous detection of specific genomic sequences targeting multiple mutations to amplify specific MLPA probes rather than target DNA [25]. In MLPA (Figure 1), two oligonucleotides (up to 45 pairs in one reaction) hybridize immediately on the target DNA. In addition to a target-specific sequence, each of the oligonucleotides contains one of two sequences recognized by a universal PCR primer pair. After denaturing and hybridization, two parts of each MLPA probe are ligated by a specific ligase enzyme, followed by PCR amplification using a universal primer pair. Non-hybridized probes are not removed, enabling a high throughput 'one-tube' method. MLPA probes are designed in such a way that each amplification product is identified by size using separation by capillary electrophoresis. Differences in relative probe signals between samples reflect differences in the probe target sequences. MLPA is widely used to identify point mutations [26], insertions [27], deletions [28], duplications [29], and recombination events [30] and is also applied for quantification of mRNAs [31] and determination of methylation status of CpG islands [32].

The elegance and simplicity of MLPA makes it applicable to various types of clinical samples, such as blood [33], amniotic fluid [34] or tumor tissue [35]. In pathogen detection, MLPA has only been applied to *Mycobacterium tuberculosis *[36], bacterial species in oral biofilms [37], respiratory viruses [38] and *Penicillium marneffei *[39], but not to parasites. In this study, we evaluate MLPA for the rapid identification of the opisthorchid liver flukes *C. sinensis*, *O. viverrini *and *O. felineus*, and establish specificity of the method to discriminate these three liver flukes in a single-tube reaction.

## Results

**Figure 2:**
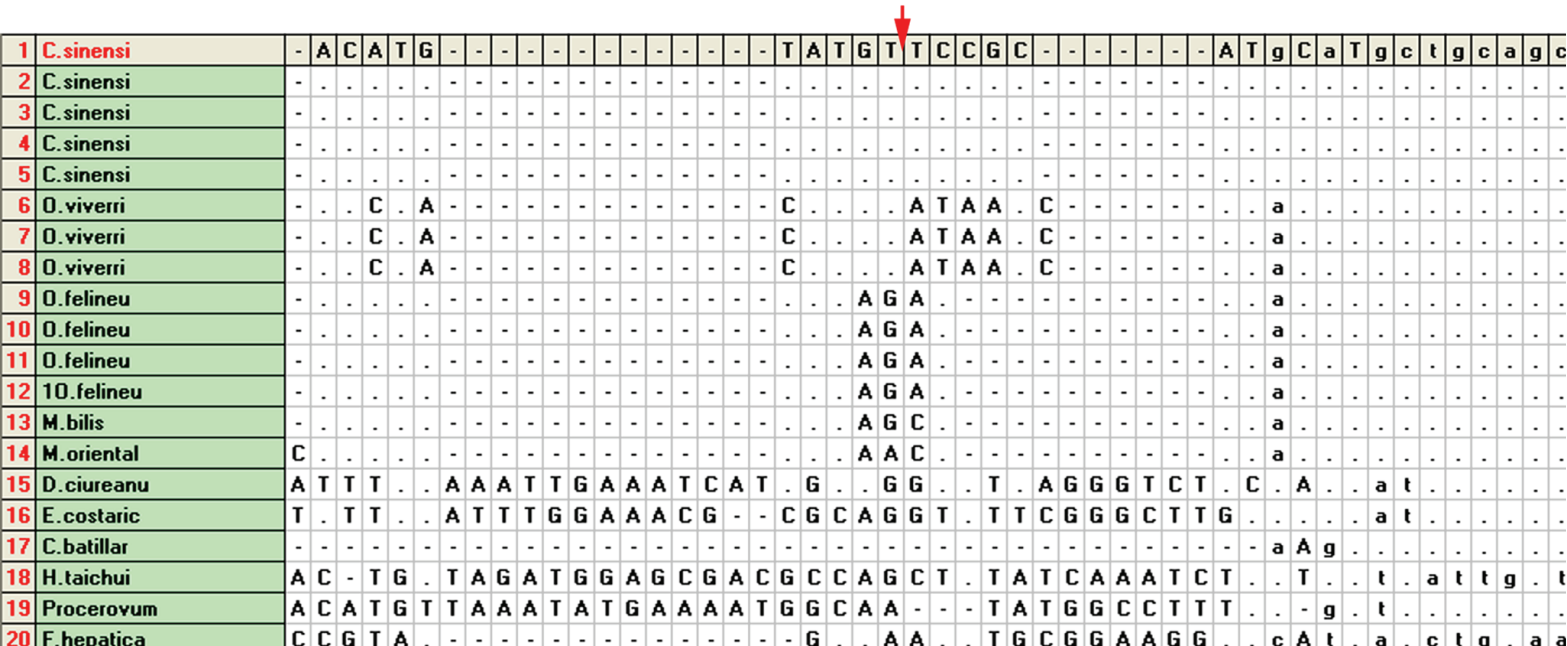
**The multiple alignment analysis of ITS1 gene of *C.sinensis, O.viverrini*, *O.felineus *and closely species in taxonomy using DNAsp software (partially)**. The arrow show the positions within nucleotides of three padlock probes designed in this study. The dots represent identical nucleotides as in the *C.sinensis *sequence given at the top, the dashes indicate deletions.

**Figure 3:**
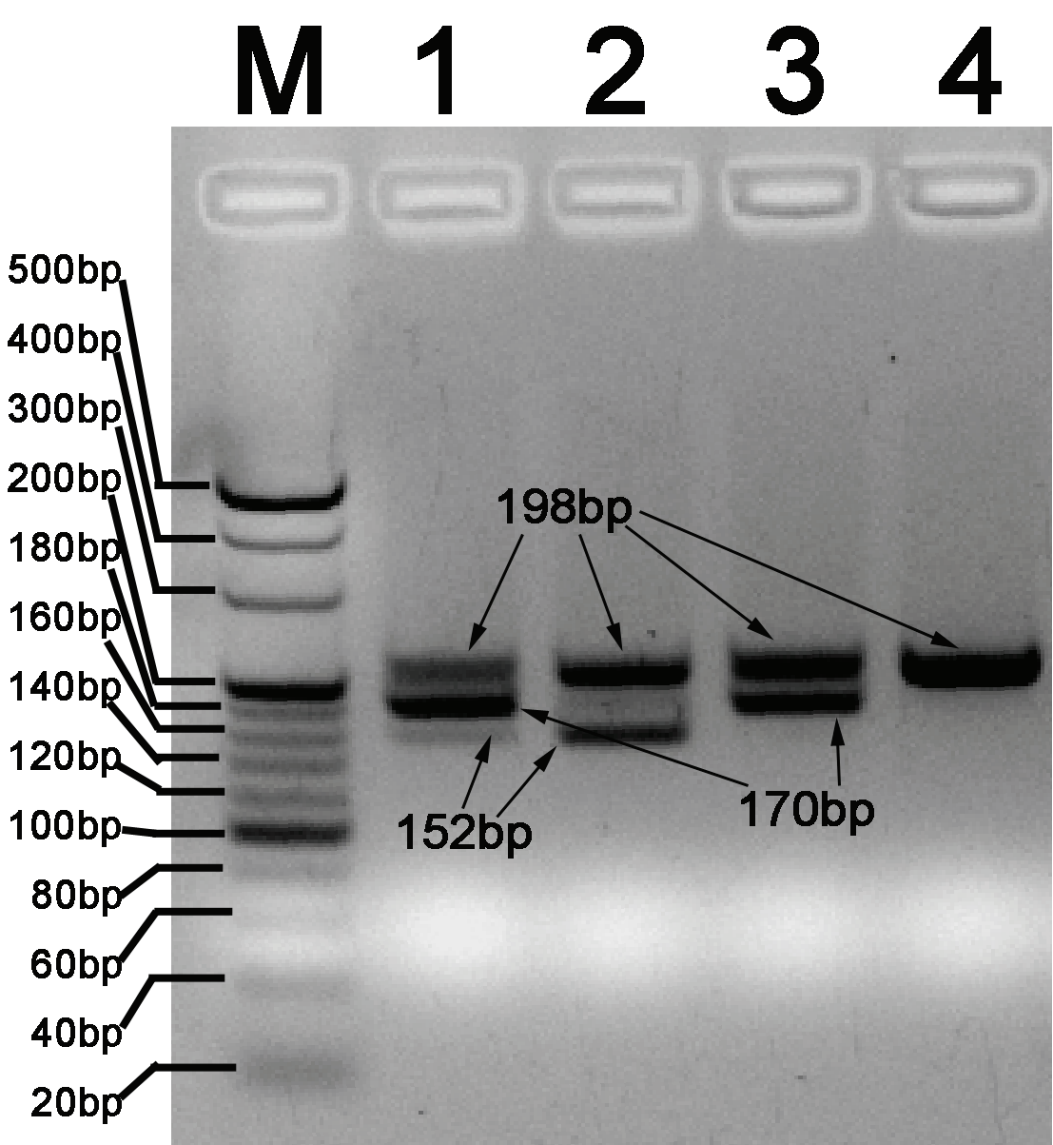
**Specificity analysis of three MLPA probes on agarose electrophoresis**. All three probe pairs (CsPL for *C.sinensis*, OfPL for *O.felineus*, OvPL for *O.viverrini*) were added, different combined artificial DNA (Cs-T for *C.sinensis*, Ov-T for *O.viverrini*, Of-T for *O.felineus*) used as templates. The MLPA amplicons were separated in 5% agarose gel by electrophoresis and the image was taken under UV light. Fragment sizes: *C.sinensis*(198bp), *O.felineus *(170bp), *O.viverrini*(152bp). Lane M, 20 bp DNA ladder; Lane1, Cs-T+Ov-T+Of-T; Lane 2, Cs-T+Ov-T; lane 3, Cs-T+ Of-T; lane 4, Cs-T.

**Figure 4:**
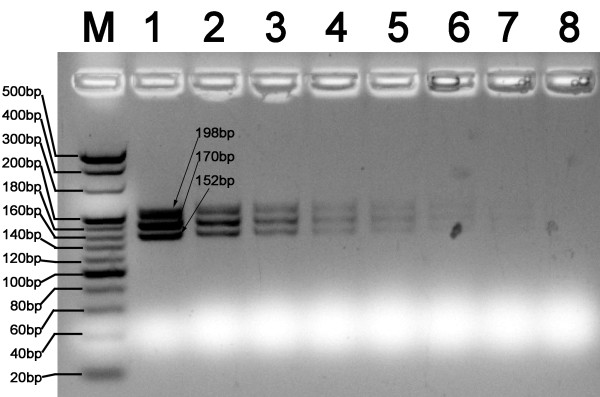
**Analytical sensitivity of MLPA assay in detection of the artificial template of the ITS1 gene**. DNA samples from these artificial templates were subjected to MLPA analysis and MLPA products were separated in 5% agarose gel by electrophoresis. M, 20 bp DNA ladder; Lanes 1 to 8, 2 × 10^9^, 2 × 10^8^, 2 × 10^7^, 2 × 10^6^, 25 × 10^5^, 2 × 10^4^, 2 × 10^3 ^and 2 × 10^2 ^copies/reaction, respectively.

**Figure 5:**
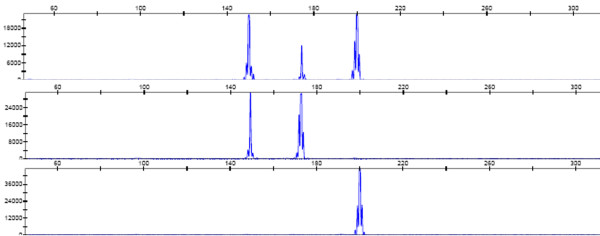
**Electropherogram showing peaks generated by MLPA**. Template DNA from individual species is used in the reaction. The MLPA reaction included all the 3 probes designed, Rox 500 as internal molecular standerd. Fragment sizes (bp) correspond to: 198 = *C.sinensis*, 170 = *O.felineus*, 152 = *O.viverrini*

In this study, the MLPA assay was adapted to identify *C. sinensis*, and discriminate with other opisthorchid liver flukes, *O. viverrini*, and *O. felineus*. We first performed a haplotype analysis of the three liver flukes and of phylogenetically related species to search for unique loci for MLPA probe design (Figure 2). Three specific loci were selected for designing species-specific pairs of oligonucleotide probes for MLPA (Table 1). Web-based BLAST analysis showed a low degree of similarity of the specific probes compared with other parasites. Three artificial templates of *C. sinensis*, *O. viverrini *and *O. felineus *were used to evaluate the specificity and sensitivity of the MLPA assay. MLPA reactions without artificial templates were used as negative controls. Artificial templates of padlock probes were used to evaluate the detection limits of the MLPA assay. These pairs of probes allowed specific amplification of the ITS1 gene of *C. sinensis*, *O. felineus *and *O. viverrini*, and yielded three amplicon of sizes 198, 170, and 152 bp, respectively (Figure 3). The amplicons were 100% consistent with the initial DNA used in each MLPA reaction. The detection limit of the MLPA assay for artificial DNA was found to be approximately 10^3 ^copies of the ITS gene for the three liver flukes analyzed (Figure 4) or 60 picogram genomic DNA for *C. sinensis *(data not shown) after the amplification products were visualized by electrophoresis on a 5% agarose gel. A total of 66 different DNA samples of adult liver flukes were tested and the data showed that the pairs of MLPA probes were able to amplify all loci presented in the samples (Table 2). No inhibition was observed after the addition of the same concentration of *Opisthorchis viverrini *or *Opisthorchis felineus *artificial template DNA (Table 2). The MLPA products could be successfully detected using a capillary sequencer (Figure 5). Moreover, specific amplification was also achieved by the use of fecal samples from infected rats (Table 3).

**Table 1:** Probes and primers used in this study

Primer	Oligonucleotides Sequences(5`-3`)
CsPLaw	P^a^***ACATACATGTAGAACATGACGG***GTTCGGGCAATTCGTTATTGGCCCTATAGTGAG*GTCTTCTCTATTGTCACCGT*ATGC**ACATCTCGGAATCAAGCTG**^g^

CsPLfw	^h^ACTGGATTCAGGTTCACGAAGCTGCATTATCGATCAGTACCAGTGTAGTACAGCGCCGGTGAAATTATCGCCACAGGCCTTT***CTGCAGCATGCATGCGGA***^b^

OfPLaw	P^c^***CTATACATGTAGAATATGACGGA***GTTCGGGCAATTCGTTATTGGCCCTATAGTGAGGTCTTCTCTATTGTCACCGTATGC**ACATCTCGGAATCAAGCTG**^g^

OfPLfw	^h^ACTGGATTCAGGTTCACGAAGCTGCATTATCGATCAGTTATCGCCACAGGCCTTT***AACGCTGCAGCATGTATGT***^d^

OvPLaw	P^e^***TTATACATGTAGGTTATACATGAC***GTTCGGGCAATTCGTTATTGGCCCTATAGTGAG*GTCTTCTCTATTGTCACCGT*ATGC**ACATCTCGGAATCAAGCTG**^g^

OvPLfw	^h^ACTGGATTCAGGTTCACGAAGCTCAGGCCTTT***AACGCTGCAGCATGTATGG***^f^

pF3	Fam-CAGCTTAGTTCCGAGATGT

pB3	ACTGGATTCAGGTTCACGA

Cs-T	CCGTCATGTTCTACATGTATGTTCCGCATGCATGCTGCAG

Of-T	TCCGTCATATTCTACATGTATAGACATACATGCTGCAGCGTT

Ov-T	GTCATGTATAACCTACATGTATAACCATACATGCTGCAGCGTT

ITS1f	CGATTCTAGTTCCGTCATCT

ITS1r	CCGCTCAGAGTTGTACTCAT

*a-f*: complementary sequence of ITS loci in padlock probes, denoted with italics and bold.

*g*: pF3 binding sequence in underline and bold.

*h*: pB3 binding sequence in underline.

P: phosphorylated

Fam: Fam fluorescent labled at the 5' end.

**Table 2:** Evaluation of the three probe pairs in 66 different strains of *C.sinensis *isolates

**No**.	GenBank	Source	CsPL+OfPL+OvPL+Ov-T+Of-T			CsPL+OfPL+OvPL+Of-T			CsPL+OfPL+OvPL		
			**198bp**	**170bp**	**152bp**	**198bp**	**170bp**	**152bp**	**198bp**	**170bp**	**152bp**

1	HQ874538	Cat, Anhui, China	+	+	+	+	+	-	+	-	-
2	HQ874523	Cat, Anhui, China	+	+	+	+	+	-	+	-	-
3	HQ874584	Cat, Anhui, China	+	+	+	+	+	-	+	-	-
4	HQ874537	Cat, Anhui, China	+	+	+	+	+	-	+	-	-
5	HQ874599	Cat, Anhui, China	+	+	+	+	+	-	+	-	-
6	HQ874585	Cat, Anhui, China	+	+	+	+	+	-	+	-	-
7	HQ874586	Cat, Anhui, China	+	+	+	+	+	-	+	-	-
8	HQ874588	Cat, Anhui, China	+	+	+	+	+	-	+	-	-
9	HQ874540	Cat, Guangdong, China	+	+	+	+	+	-	+	-	-
10	HQ874535	Cat, Guangdong, China	+	+	+	+	+	-	+	-	-
11	HQ874541	Cat, Guangdong, China	+	+	+	+	+	-	+	-	-
12	HQ874602	Cat, Guangdong, China	+	+	+	+	+	-	+	-	-
13	HQ874587	Cat, Guangdong, China	+	+	+	+	+	-	+	-	-
14	HQ874532	Cat, Guangdong, China	+	+	+	+	+	-	+	-	-
15	HQ874581	Cat, Guangdong, China	+	+	+	+	+	-	+	-	-
16	HQ874582	Cat, Guangdong, China	+	+	+	+	+	-	+	-	-
17	HQ874542	Cat, Guangxi, China	+	+	+	+	+	-	+	-	-
18	HQ874536	Cat, Guangxi, China	+	+	+	+	+	-	+	-	-
19	HQ874543	Cat, Guangxi, China	+	+	+	+	+	-	+	-	-
20	HQ874529	Cat, Guangxi, China	+	+	+	+	+	-	+	-	-
21	HQ874580	Cat, Guangxi, China	+	+	+	+	+	-	+	-	-
22	HQ874533	Cat, Guangxi, China	+	+	+	+	+	-	+	-	-
23	HQ874525	Cat, Guangxi, China	+	+	+	+	+	-	+	-	-
24	HQ874579	Cat, Guangxi, China	+	+	+	+	+	-	+	-	-
25	HQ874544	Cat, Hubei, China	+	+	+	+	+	-	+	-	-
26	HQ874545	Cat, Hubei, China	+	+	+	+	+	-	+	-	-
27	HQ874593	Cat, Hubei, China	+	+	+	+	+	-	+	-	-
28	HQ874578	Cat, Hubei, China	+	+	+	+	+	-	+	-	-
29	HQ874539	Cat, Hubei, China	+	+	+	+	+	-	+	-	-
30	HQ874592	Cat, Hubei, China	+	+	+	+	+	-	+	-	-
31	HQ874546	Cat, Hubei, China	+	+	+	+	+	-	+	-	-
32	HQ874547	Cat, Hubei, China	+	+	+	+	+	-	+	-	-
33	HQ874524	Cat, Hubei, China	+	+	+	+	+	-	+	-	-
34	HQ874601	Cat, Henan, China	+	+	+	+	+	-	+	-	-
35	HQ874550	Cat, Henan, China	+	+	+	+	+	-	+	-	-
36	HQ874597	Cat, Henan, China	+	+	+	+	+	-	+	-	-
37	HQ874595	Cat, Henan, China	+	+	+	+	+	-	+	-	-
38	HQ874573	Cat, Henan, China	+	+	+	+	+	-	+	-	-
39	HQ874572	Cat, Henan, China	+	+	+	+	+	-	+	-	-
40	HQ874571	Cat, Henan, China	+	+	+	+	+	-	+	-	-
41	HQ874589	Cat, Henan, China	+	+	+	+	+	-	+	-	-
42	HQ874598	Cat, Hunan, China	+	+	+	+	+	-	+	-	-
43	HQ874590	Cat, Hunan, China	+	+	+	+	+	-	+	-	-
44	HQ874591	Cat, Hunan, China	+	+	+	+	+	-	+	-	-
45	HQ874551	Cat, Hunan, China	+	+	+	+	+	-	+	-	-
46	HQ874534	Cat, Hunan, China	+	+	+	+	+	-	+	-	-
47	HQ874552	Cat, Hunan, China	+	+	+	+	+	-	+	-	-
48	HQ874553	Cat, Hunan, China	+	+	+	+	+	-	+	-	-
49	HQ874554	Cat, Hunan, China	+	+	+	+	+	-	+	-	-
50	HQ874555	Dog, Jilin, China	+	+	+	+	+	-	+	-	-
51	HQ874556	Dog, Jilin, China	+	+	+	+	+	-	+	-	-
52	HQ874557	Dog, Jilin, China	+	+	+	+	+	-	+	-	-
53	HQ874570	Dog, Jilin, China	+	+	+	+	+	-	+	-	-
54	HQ874528	Dog, Jilin, China	+	+	+	+	+	-	+	-	-
55	HQ874527	Dog, Jilin, China	+	+	+	+	+	-	+	-	-
56	HQ874558	Cat, Jiangsu, China	+	+	+	+	+	-	+	-	-
57	HQ874566	Cat, Jiangsu, China	+	+	+	+	+	-	+	-	-
58	HQ874559	Cat, Jiangsu, China	+	+	+	+	+	-	+	-	-
59	HQ874530	Cat, Jiangsu, China	+	+	+	+	+	-	+	-	-
60	HQ874583	Cat, Jiangsu, China	+	+	+	+	+	-	+	-	-
61	HQ874569	Cat, Jiangsu, China	+	+	+	+	+	-	+	-	-
62	HQ874604	Cat, Jiangsu, China	+	+	+	+	+	-	+	-	-
63	HQ874565	Cat, Jiangxi, China	+	+	+	+	+	-	+	-	-
64	HQ874560	Cat, Jiangxi, China	+	+	+	+	+	-	+	-	-
65	HQ874561	Cat, Jiangxi, China	+	+	+	+	+	-	+	-	-
66	HQ874564	Cat, Jiangxi, China	+	+	+	+	+	-	+	-	-
67		Negative control	-	-	-	-	-	-	-	-	-

CsPL: *Clonorchis sinensis *Padlock probe pairs

OfPL: *Opisthorchis felineus *Padlock probe pairs

OvPL: *Opisthorchis viverrini *Padlock probe pairs

Cs-T: Artificial template of *Clonorchis sinensis *Padlock probe pairs

Ov-T: Artificial template of *Opisthorchis viverrini *Padlock probe pairs

Of-T: Artificial template of *Opisthorchis felineus *Padlock probe pairs

**Table 3:** Fecal samples of infected rats and data from the three different detection methods

	Detection method					
	
**Fecal Samples no**.	Microscopy detection	ITS PCR		MLPA		
		
		First Run	Second Run	CsPL	OvPL	OfPL
1	**+**	-	**+**	**+**	-	-
2	**+**	-	**+**	**+**	-	-
3	**+**	-	**+**	**+**	-	-
4	**+**	-	**+**	**+**	-	-
5	**+**	-	**+**	**+**	-	-
6	**+**	-	**+**	**+**	-	-
7	**+**	-	**+**	**+**	-	-
8	**+**	-	**+**	**+**	-	-
9	**+**	-	**+**	**+**	-	-
10	**+**	-	**+**	**+**	-	-
11	**+**	-	**+**	**+**	-	-
12	**+**	-	**+**	**+**	-	-
13	**+**	-	**+**	+	-	-
14	**+**	-	**+**	**+**	-	-
15	**+**	-	**+**	**+**	-	-
16	**+**	-	**+**	**+**	-	-
17	**+**	-	**+**	**+**	-	-
18	**+**	-	**+**	**+**	-	-
19	**+**	-	**+**	**+**	-	-
20	**+**	-	**+**	**+**	-	-
21	**+**	-	**+**	**+**	-	-
22	**+**	-	**+**	**+**	-	-
23	**+**	-	**+**	**+**	-	-
24	**+**	-	**+**	**+**	-	-
25	**+**	-	**+**	+	-	-
26	**+**	-	**+**	+	-	-
27	**+**	-	**+**	**+**	-	-
28	**+**	-	**+**	**+**	-	-
29	**+**	-	**+**	**+**	-	-
30	**+**	-	**+**	**+**	-	-
31	**+**	-	**+**	**+**	-	-
32	**+**	-	**+**	**+**	-	-
33	**+**	-	**+**	**+**	-	-
34	**+**	-	**+**	**+**	-	-
35	**+**	-	**+**	**+**	-	-
36	**+**	-	**+**	**+**	-	-
37	-	-	-	-	-	-
38	-	-	-	-	-	-
39	-	-	-	-	-	-
40	-	-	-	-	-	-
41	-	-	-	-	-	-
42	-	-	-	-	-	-
43	-	-	-	-	-	-
44	-	-	-	-	-	-
45	-	-	-	-	-	-
46	-	-	-	-	-	-
47	-	-	-	-	-	-
48	-	-	-	-	-	-

CsPL: *Clonorchis sinensis *Padlock probe pairs

OfPL: *Opisthorchis felineus *Padlock probe pairs

OvPL: *Opisthorchis viverrini *Padlock probe pairs

## Discussion

The use of ligated oligonucleotide probes in MLPA, and a number of other methods, allows specific detection of changes in single nucleic acids of targeted genes [40-42]. These probes hybridize to and capture these areas, which are then enriched through rolling circle amplification or by PCR. Using these probes we were able to simultaneously detect gene loci and characterize different strains in a single reaction. In the present study we evaluate the MLPA assay to identify and discriminate three opisthorchid liver flukes. The sensitivity and specificity of MLPA highlights that the method is a useful tool for prompt and accurate diagnosis, pathogen characterization, and epidemiological studies of parasite infections.

In our initial test, we evaluated the MLPA assay to identify and discriminate three liver flukes using artificial templates. All of the three probe pairs used allow specific amplification of the ITS1 locus of each respective species. The MLPA reaction was sensitive enough to detect 10^3 ^copies of artificial template DNA. This is consistent with previous studies on MLPA in oral biofilm, where DNA was detected at picogram levels [37]. The size of the *C. sinensis *genome varies from 500 to 700 Mbp (Wang et al., unpublished data), and 1 pg of DNA is equal to 978 Mbp of genomic DNA [43]. The weight of *C. sinensis *DNA is approximately 0.511-0.716 pg and the 10^3 ^copies of *C. sinensis *DNA detectable by MLPA is then roughly equivalent to 0.5-70 pg of genomic DNA. These results were consistent with the 60 pg genomic DNA of *C. sinensis *mentioned above. Results were comparable to a previous study indicating that the sensitivity of MLPA is equivalent to real-time PCR [38], while similar results were obtained in some studies focusing on *O. viverrini *[24] and *C. sinensis *[18]. However, our results deviate from a previous report where with simple PCR a detection limit of 10^-12 ^ng was obtained [17]. This might be explained by the use of different target genes or by different copy numbers of target genes in the genome.

Furthermore, the results of the inhibition test indicated that the MLPA assay was not inhibited by the presence of non-target DNA. These data demonstrate a considerable potential for MLPA in future clinical applications, which normally involve complex DNA mixtures. Although enhancement of sensitivity requires further optimization to capture low copy numbers of template DNA, the alternative strategy would be to increase the efficiency of the MLPA reaction, or the employment of more sensitive detection equipment. The first option might be achieved by the addition of more efficient amplification facilitators such as dimethyl sulfoxide [44], dithiothreitol [45], betaine [46], bovine serum albumin and single-stranded DNA binding T4 gene 32 protein (gp32) [47]. For the later option, a real time detector could be used to track the limited fluorescent-labeled amplicons [38]. The results would be comparable with those of capillary electrophoresis or of fragment analysis of fluorescent-labeled amplicons. However, the electrophoresis maybe the optimized method to detect MLPA products for unequipped laboratory or lab of local hospital [25,48].

## Conclusion

In the current study the MLPA assay was adapted to identify and discriminate three liver flukes in a 'one-tube' reaction, which was proven to be a sensitive and specific tool with high efficiency. Multiplex amplification makes this assay useful for high through-put analysis of pathogens in large clinical or ecological samples [48]. The flexible arms of the probes allow for minimal inflorescent labeling. The advantages of this method have a potential for wider application, e.g. to the detection of other parasites or to diagnostics of mixed infections in severely ill patients.

## Material and Methods

### Ethical Standards

All animals were handled in strict accordance with good animal practice as defined by the relevant national and/or local animal welfare bodies. Procedures involving vertebrate animals were reviewed and approved by Sun Yat-Sen University's Animal Care and Use Committee.

### Parasite sampling and genomic DNA extraction

Sixty-six *C. sinensis *individuals were collected from infected cats or dogs, the most common reservoir hosts, in 9 provinces in China mainland (Table 4). Genomic DNA from adult worms was extracted using a commercial DNA extraction kit (Dong sheng Biocompany, Guangdong, China) according to manual instruction. Briefly as: single adult was ground in a 1.5 ml microcentrifuge tube containing 200 μl of extraction buffer I, after shortly homogenizing, proteinase K(New England Biolabs, U.K.) and RNase A(New England Biolabs, U.K.) were added to final concentrations of 100 μg/ml and 20 μg/ml, respectively, and incubated for 3 h at 37°C. Following this, 200 μl Buffer II was added to the mixture, and incubated for 10 min at 65°C. Then, 200 μl ethanol was added to the mixture. Totally mixture was moved into the spin column after tightly vortex, after spin for 1 min at 8000 rpm, extra protein was removed using Buffer III, and then the column was washed twice with 70% ethanol, followed by centrifuge at 12000 rpm for 2 min to remove extra ethanol, DNA was recovery using 50 μl buffer EB. RNAse (5 μl each, 10 mg/ml in pH7.4 NaAC) treatment was performed at 37°C for 30 min. The DNA quantification was done at 260 nm in a UV spectrophotometer (Shimadzu, Japan).

**Table 4:** *C.sinensis *isolates and strain information of reference ITS gene sequences in this study

Species	GenBank	Source
*Clonorchis sinensis*	EU038112	Shenyang, China
	EU038113	Shenyang, China
	EU038114	Shenyang, China
	EU038115	Shenyang, China
	EU038116	Shenyang, China
	EU038117	Shenyang, China
	EU038118	Shenyang, China
	EU038119	Shenyang, China
	EU038120	Gimhae-si, Gyeongsangnam-do, South Korea
	EU038121	Gimhae-si, Gyeongsangnam-do, South Korea
	EU038122	Gimhae-si, Gyeongsangnam-do, South Korea
	EU038123	Gurye-gun, Jeollanam-do, SouthKorea
	EU038124	Gurye-gun, Jeollanam-do, SouthKorea
	EU038125	Gurye-gun, Jeollanam-do, SouthKorea
	EU038126	Jinju-si, Gyeongsangnam-do, South Korea
	EU038127	Jinju-si, Gyeongsangnam-do, South Korea
	EU038128	Jinju-si, Gyeongsangnam-do, South Korea
	EU038129	Jinju-si, Gyeongsangnam-do, South Korea
	EU038130	Jinju-si, Gyeongsangnam-do, South Korea
	EU038131	Jinju-si, Gyeongsangnam-do, South Korea
	HQ874538	Cat, Anhui, China
	HQ874523	Cat, Anhui, China
	HQ874584	Cat, Anhui, China
	HQ874537	Cat, Anhui, China
	HQ874599	Cat, Anhui, China
	HQ874585	Cat, Anhui, China
	HQ874586	Cat, Anhui, China
	HQ874588	Cat, Anhui, China
	HQ874540	Cat, Guangdong, China
	HQ874535	Cat, Guangdong, China
	HQ874541	Cat, Guangdong, China
	HQ874602	Cat, Guangdong, China
	HQ874587	Cat, Guangdong, China
	HQ874532	Cat, Guangdong, China
	HQ874581	Cat, Guangdong, China
	HQ874582	Cat, Guangdong, China
	HQ874542	Cat, Guangxi, China
	HQ874536	Cat, Guangxi, China
	HQ874543	Cat, Guangxi, China
	HQ874529	Cat, Guangxi, China
	HQ874580	Cat, Guangxi, China
	HQ874533	Cat, Guangxi, China
	HQ874525	Cat, Guangxi, China
	HQ874579	Cat, Guangxi, China
	HQ874544	Cat, Hubei, China
	HQ874545	Cat, Hubei, China
	HQ874593	Cat, Hubei, China
	HQ874578	Cat, Hubei, China
	HQ874539	Cat, Hubei, China
	HQ874592	Cat, Hubei, China
	HQ874546	Cat, Hubei, China
	HQ874547	Cat, Hubei, China
	HQ874524	Cat, Hubei, China
	HQ874601	Cat, Henan, China
	HQ874550	Cat, Henan, China
	HQ874597	Cat, Henan, China
	HQ874595	Cat, Henan, China
	HQ874573	Cat, Henan, China
	HQ874572	Cat, Henan, China
	HQ874571	Cat, Henan, China
	HQ874589	Cat, Henan, China
	HQ874598	Cat, Hunan, China
	HQ874590	Cat, Hunan, China
	HQ874591	Cat, Hunan, China
	HQ874551	Cat, Hunan, China
	HQ874534	Cat, Hunan, China
	HQ874552	Cat, Hunan, China
	HQ874553	Cat, Hunan, China
	HQ874554	Cat, Hunan, China
	HQ874555	Dog, Jilin, China
	HQ874556	Dog, Jilin, China
	HQ874557	Dog, Jilin, China
	HQ874570	Dog, Jilin, China
	HQ874528	Dog, Jilin, China
	HQ874527	Dog, Jilin, China
	HQ874558	Cat, Jiangsu, China
	HQ874566	Cat, Jiangsu, China
	HQ874559	Cat, Jiangsu, China
	HQ874530	Cat, Jiangsu, China
	HQ874583	Cat, Jiangsu, China
	HQ874569	Cat, Jiangsu, China
	HQ874604	Cat, Jiangsu, China
	HQ874565	Cat, Jiangxi, China
	HQ874560	Cat, Jiangxi, China
	HQ874561	Cat, Jiangxi, China
	HQ874564	Cat, Jiangxi, China
*Opisthorchis felineus*	EU038134	Novosibirsk, Russia
	EU038135	Novosibirsk, Russia
	EU038136	Novosibirsk, Russia
	EU038137	Novosibirsk, Russia
	EU038138	Novosibirsk, Russia
	EU038139	Novosibirsk, Russia
	EU038140	Novosibirsk, Russia
*Opisthorchis viverrini*	EU038150	Vientiane, Laos
	EU038151	Vientiane, Laos
	EU038152	Vientiane, Laos
	EU038153	Vientiane, Laos
	EU038141	Khammouan, Laos
	EU038142	Khammouan, Laos
	EU038143	Khammouan, Laos
	EU038144	Savannakhet, Laos
	EU038145	Savannakhet, Laos
	EU038146	Savannakhet, Laos
	EU038147	Savannakhet, Laos
	EU038148	Savannakhet, Laos
*Metorchis bilis*	EU038154	Spain
*Metorchis orientalis*	HM347228	Pseudorasbora parva, China
*Dexiogonimus ciureanus*	AY245702	Israel
*Euryhelmis costaricensis*	AB521800	Aomori, Nishimeya Village, Japan
*Procerovum sp.*	AB536892	fish-metacercaria, Nakorn Pathom, Thailand
*Haplorchis taichui*	AB536889	fecal sample, Savannakhet, Laos
*Cercaria batillariae*	AY626543	Miyagi, Japan
*Fasciola hepatica*	FJ756394	Iran

### Fecal sampling, DNA extraction and qualification

Thirty-six fecal samples were collected from 36 infected rats at 8 weeks after infection with metacercariae. Twelve fecal samples from 12 uninfected rats were used as control. The feces of rats were firstly examined by FECT methods[49]. One gram of feces was taken for FECT. The pellet after centrifugation was resuspended with 1 ml of 10% formalin and 20 μl of suspension was used for microscopy detection.

DNA was extracted from fecal samples as described previously[50]. Briefly, 800 mg feces were washed twice with 1 ml PBS. After centrifugation, the pellet was resuspended into 200 μl of 2% polyvinylpolypyrolidone (PVPP, Sigma, St. Louis, MO) and heated for 10 min at 100°C. After sodium dodecyl sulfate-proteinase K treatment (2 h at 55°C), DNA was isolated using QIAamp Tissue Kit spin columns (QIAgen, Hilden, Germany), and eluted using 100 μl of elution buffer.

The quality of the DNA of *C.sinensis *was confirmed by successful PCR amplification with universal fungal primers ITS1F and ITS1R [51]. The first run of PCR to detect fungal DNA was performed as follows: an initial 95°C for 5 min and then 25 cycles of 95°C for 30 s, 62°C for 30 s, 72°C for 1 min, and a final extension at 72°C for 7 min. Then one microliter amplicons of first run was used as the PCR template for the second run under the same reaction program. Amplicons were analyzed by electrophoresis (Bio-Rad, Hercules, CA) on 2% agarose gels (NuSieve, Rockland, ME).

### Nucleotide polymorphism analysis of ITS gene of C.sinensis isolates

ITS1 rDNA regions of *C.sinensis *were amplified using primers ITS1F and ITS1R [51]. PCR was performed as follows: 95°C for 5 min; 25 cycles of 95°C for 30 s, 62°C for 30 s, 72°C for 1 min, with final extension at 72°C for 7 min. Amplicons were detected by electrophoresis (Bio-Rad, California, U.S.A.) on 2% agarose gel (NuSieve, Rockland, ME U.S.A.), then amplicons were sequenced with primer ITS1Fand ITS1R by Invitrogen company (Invitrogen, Shanghai, China). Sequences were edited using SEQMAN in the Lasergene software (DNASTAR, Wisconsin, U.S.A.) and submitted to GenBank. Sequences of ITS1 of *O. viverrini*, *O.felineus *and other references strains closely to liver flukes in phylogenetic were down load from GenBank. All sequences aligned with Bionumerics version 4.61 (Applied Maths, Kortrijk, Belgium). Single-nucleotide polymorphism of ITS1 gene were analyzed by DNaSP4 software (Universitat de Barcelona; Software for Population Genetics and Molecular Evolution analyse; 4.20.0002).

### Probe design

DNA sequences of *C.sinensis*, *O. viverrini*, *O.felineus *and reference strains were aligned automatically and adjusted manually in BioNumerics v. 4.61 (Applied Maths, Kortrijk, Belgium) to identify informative nucleotide polymorphisms. The design of the MLPA probes was performed as described [25]. Three pairs of completely synthetic MLPA probes targeting the ITS region were designed with an annealing temperature >65°C according to the RAW program (http://www.mlpa.com/WebForms/WebFormMain.aspx ) and no secondary structures according to the mFOLD server (http://www.bioinfo.rpi.edu/applications/mfold). For probe sequences were listed in Table 2. Specificity of the probes was confirmed by BLAST analysis in GenBank.

### MLPA analysis

MLPA reactions were performed referring to the standard protocol on http://www.mlpa.com/WebForms/WebFormMain.aspx?Tag=wl2zCji\rCGANQgZPuTixtCplCA1mmwJoFo/xHPnTgc| with some modifications. Briefly as: Routinely 500 pg of DNA from pure culture was used. All the MLPA reagents come from MRC-Holland (Amsterdam the Netherlands). The hybridization and ligation of probes were performed in Biosystems 2720 thermal cycler according to the standard MLPA protocol. pF3 (Fam fluorescent labeled at 5' end) and pB3 were used as universal PCR primers in the ligated probes amplification. PCR amplification was performed for 25 cycles (30 s 95°C, 30 s 55°C and 1 min 72°C) with a denature at 95°C for 5 min and a extension step at 72°C for 7 min.

### Specificity and validation of signal quantification

Genome DNA of 66 *C.sinensis *and 3 artificial template of *C.sinensis*, *O. viverrini *and *O.felineus *were used as templates to evaluate the specificity and sensitivity of the MLPA assay. MLPA reaction without *C.sinensis *DNA or artificial templates were used as negative controls. Artificial template of padlock probe was used to evaluate the detection limit of the MLPA assay. Two microlitres of each 10-fold serial diluted artificial template mixture and genome DNA of *C.sinensis *was used as templates for MLPA reaction. Amplified products were analyzed by electrophoresis on 5% agarose gels, stained with ethidium bromide and photographed. 20bp DNA ladder was used as molecular weight standard.

### Results detection using capillary sequencer

One microliter of the products was dissolved in 9 μl of deionized formamide, 0.2 nM Gene-Scan^®^-ROX 500 size standards, and 0.5 μl loading dye (all from Applied Biosystems, Foster City, CA, USA) and denatured for five minutes at 95°C. The products were electrophoresed on an ABI Prism^® ^3730XL Genetic Analyzer model capillary sequencer (Applied Biosystems) in the GeneScan mode. Analysis of the products was performed using Gene-Scan 3.7 and Genotyper^® ^3.7 software (Applied Biosystems) consecutively.

### Evaluation of MLPA in fecal samples of infected rats

Crude-extracted DNA of 2 μl each from 48 fecal samples was used as a template for MLPA assays. The amplified products were analyzed by electrophoresis.

## Competing interests

The authors declare that they have no competing interests.

## Authors' contributions

XY, JX and JS designed the present experiments. JS carried out these experiments and drafted the manuscript. PL, QM and CL collected the isolates using in this study. YH, XL and CD give crucial reviews of this manuscript, GSdH give crucial English revision to this manuscript. All authors read and approved the final version of the manuscript.
